# Integrated Microbiome and Metabolome Analysis Reveals a Positive Change in the Intestinal Environment of *Myostatin* Edited Large White Pigs

**DOI:** 10.3389/fmicb.2021.628685

**Published:** 2021-02-17

**Authors:** Yangli Pei, Chujie Chen, Yulian Mu, Yalan Yang, Zheng Feng, Bugao Li, Hua Li, Kui Li

**Affiliations:** ^1^Guangdong Provincial Key Laboratory of Animal Molecular Design and Precise Breeding, Key Laboratory of Animal Molecular Design and Precise Breeding of Guangdong Higher Education Institutes, School of Life Sciences and Engineering, Foshan University, Foshan, China; ^2^Institute of Animal Sciences, Chinese Academy of Agricultural Sciences, Beijing, China; ^3^College of Animal Science, Shanxi Agricultural University, Shanxi, China

**Keywords:** *myostatin*, microbiome, metabolome, jejunum, cecum, pigs

## Abstract

Myostatin (MSTN) functional inactivation can change the proportion of lean meat and fat content in pigs. While both genotype and microbial composition are known to affect the host phenotype, so far there has been no systematic study to detect the changes in the intestinal microbial composition and metabolome of *MSTN* single copy mutant pigs. Here, we used 16S rDNA sequencing and metabolome analysis to investigate how *MSTN* gene editing affects changes in the microbial and metabolome composition in the jejunum and the cecum of Large White pigs. Our results showed that *Clostridium_sensu_stricto_1*, *Bifidobacterium*, *Lachnospiraceae_UCG-007*, *Clostridium_sensu_stricto_6*, *Ruminococcaceae_UCG-002*, and *Ruminococcaceae_UCG-004* were significantly upregulated; while *Treponema_2* and *T34_unclassified* were significantly downregulated in the jejunum of MSTN pigs. Similarly, *Phascolarctobacterium*, *Ruminiclostridium_9*, *Succinivibrio*, *Longibaculum*, and *Candidatus_Stoquefichus* were significantly upregulated, while *Barnesiella* was significantly downregulated in the cecum of MSTN pigs. Moreover, metabolomics analysis showed significant changes in metabolites involved in purine, sphingolipid and tryptophan metabolism in the jejunum, while those associated with glycerophospholipid and pyrimidine metabolism were changed in the cecum. Spearman correlation analysis further demonstrated that there was a significant correlation between microflora composition and metabolites. Our analyses indicated the *MSTN* editing affects the composition of metabolites and microbial strains in the jejunum and the cecum, which might provide more useable nutrients for the host of *MSTN*± Large White pigs.

## Introduction

Myostatin (MSTN) is a protein that inhibits muscle development (McPherron et al., 1997) and its inactivation interferes with fat deposition, which results in a higher proportion of lean meat (Zhao et al., 2005; Guo et al., 2009). Pigs are a major source of high-value animal protein, whereby farming animals of containing with *MSTN* loss-of-function mutations has become a research priority to facilitate the breeding of stock with better meat quality and higher economic value. Previous studies have reported the successful generation of healthy *MSTN* knockouts in different pig breeds, including Meishan (Qian et al., 2015), Erhualian (Wang et al., 2017), Large White/Landrace × Duroc (Rao et al., 2016) and Landrace pigs (Wang et al., 2015). The animals showed a reduced fat content and increased tenderness. Furthermore, pigs harboring naturally occurring single copy *MSTN* mutations have higher muscle mass and lower fat content compared to the wild-type genotype (Matika and Robledo, 2019). In fact, the phenotype of mutant MSTN pigs is affected by both double and single mutations that consistently the host leaner, with higher muscle and lower fat proportions. Therefore, these genotypes are likely to have significant future applications.

On the other hand, the gut microbial community is a complex system that co-exists inside each living body and associated with meat quality and body fat (Park et al., 2014). Yang et al. (2017) reported that microbial transplantation from pigs to mice changed the metabolic profiles of skeletal muscle (Yang et al., 2017). Mach et al. (2015) investigated the early establishment of the microbiome in pigs and identified enterotypes related to growth (Ramayo-Caldas et al., 2016), and (Xiao et al., 2016) reported the potential effects of different microbial profiles on lipid metabolism. Finally, Lu et al. (2018) reported a relationships between growth and carcass composition and specific microbial composition as well as alpha diversity (Lu et al., 2018). These observations demonstrate that gut bacteria affect host nutritional, physiological and immunological processes in various ways (Maltecca and Bergamaschi, 2020). Indeed, interference with intestinal microbial homeostasis is known to have downstream effects on intestinal metabolism (Alou et al., 2016; Lippert et al., 2017).

Given the above literatures, *MSTN* gene mutation can affect the proportion of lean meat and fat of pigs. And the gut microbial is associated with meat quality and body fat Nevertheless, up to data there is no a systematic study detecting the changes in intestinal microbial composition and metabolome of *MSTN* single mutant pigs. In this study, we used 16S rDNA gene sequencing and metabolome analysis to investigate changes in the microbial composition and metabolome in the jejunum and the cecum of *MSTN* edited Large White pigs.

## Materials and Methods

### Animals

The pigs had *ad libitum* access to a commercial pig diet and water throughout the study period. All experiments involving animals were approved by the Animal Welfare and Research Ethics Committee at the Institute of Animal Sciences, Chinese Academy of Agricultural Sciences (IAS2018-10). Animal care and treatment were complied with the standards described in the guidelines for the care and use of laboratory animals of the Institute of Animal Sciences of CAAS.

### Sampling

To minimize the number of variables in the experiment, we selected six heterozygous mutants (average body weight: 114.4441 ± 3.6756 kg; average body length: 100.2235 ± 0.9389 cm) and six wild type (average body weight: 115.3689 ± 3.6499 kg; average body length: 114.4441 ± 3.6756 cm) 8 month old pigs from the offspring of the same *MSTN*^+/−^ mutant boars (Supplementary Table 1). The animals were raised on the same farm with the same management. Feed came from Tianjin Taikang feed mill, and contained corn, fish meal, soybean meal, sodium chloride, amino acids, vitamins, trace elements, stone powder and calcium hydrogen phosphate; the nutrient levels of the diets can be found in Supplementary Table 2. The animals were electrocuted, and the jejunum and cecum contents were immediately removed.

### Genotype Identification

Samples of piglets’ ear tissue were taken and preserved in alcohol. Total DNA was extracted according to the instructions of the animal tissue genomic DNA extraction Kit (TIANGEN, DP324). After that, the *MSTN* gene was amplified with 2 × Es Taq MasterMix (CoWin Biosciences). The PCR amplification products of *MSTN* (20 μL) were detected by 1.5% agar-gel electrophoresis. The PCR products (20 μL) were sequenced by Sangon Biotech (Shanghai) Co., Ltd.

### 16S rDNA Sequencing

DNA from fecal samples was isolated using the Stool DNA Kit (Omega, United States). The primers (F: 5′-ACTCCTACGGGAGGCAGCAG-3′; R: 5′-GGACTACHVGGGTWT-CTAAT-3′) were used in the PCR amplification of the V3–V4 region of the bacterial 16S rRNA gene. The 5′ ends of the primers were tagged with specific barcods per sample and sequencing universal primers. PCR amplification was performed in a total volume of 25 μL reaction mixture containing 25 ng of template DNA, 12.5 μL PCR Premix, 2.5 μL of each primer, and PCR-grade water. The size of the PCR products was confirmed with 2% agarose gel electrophoresis, purified with AMPure XT beads (Beckman Coulter Genomics, Danvers, MA, United States) and then quantified by Qubit (Invitrogen, CA, United States). The amplicon pools were prepared for sequencing, and the size and quantity of the amplicon library were assessed on an Agilent 2100 bioanalyzer (Agilent, United States) and with the Library Quantification Kit for Illumina (Kapa Biosciences, Woburn, MA, United States).

Amplicon libraries were sequenced on an Illumina MiSeq platform according to the manufacturer’s recommendations, which were provided by LC-Bio. Feature list and feature sequence were obtained by removing the background. Non-metric Multidimensional Scaling(NMDS) analysis was performed according to the weighted UniFrac distance metrics. The number of observed species and the indices of Chao 1 (species richness), Shannon and Simpson (diversity) were calculated to estimate alpha diversity. The PICRUSt software was used to predict the function of composition of samples.

The dataset for 16S rDNA sequencing was deposited in GenBank Sequence Read Archive (SRA) database and are available under the accession number PRJNA687099 (https://dataview.ncbi.nlm.nih.gov/object/PRJNA687099).

### Untargeted Metabolomic Study

All fecal samples were thawed on ice, and metabolite were extracted with 50% methanol Buffer. The detailed extraction procedures can be found in (Yu et al., 2018). LC-MS analysis was performed on an ultra performance liquid chromatography (UPLC) system (SCIEX, United Kingdom) coupled with the high-resolution tandem mass spectrometer TripleTOF5600plus (SCIEX, United Kingdom). The Q-TOF was operated in both positive and negative ion modes.

The raw LC-MS data files were converted into mzXML format, and then processed by the XCMS, CAMERA and metaX (Li et al., 2018) toolbox, which was implemented in R. The online KEGG and HMDB databases were used to annotate the metabolites by matching the exact molecular mass data (m/z) of the samples with those from the database.

Student *t*-tests were used to calculate significant differences in metabolite concentrations between the two groups. We have implemented multiple test corrections by adjusting the *P* values using an FDR (Benjamini–Hochberg). Supervised PLS-DA was conducted through metaX in order to discriminate the different variables between groups. The metabolites with VIP > 1, *P*-value < 0.05 and fold change(FC) ≥ 1.5 or FC ≤ 0.5 were considered significantly different. Furthermore, significantly differentially abundant metabolites screened from untargeted metabolomics were imported into the MetaboAnalyst 4.0 database to perform pathway analysis.

### Correlation Analysis

We performed spearman correlation analysis on the differentiated metabolites screened by metabolomics and the significantly different genera obtained by 16S rDNA sequencing analysis.

## Results

### Genotype Identification

The pigs used in this study were selected from the offspring of healthy MSTN^+/−^ mutant boars created by removing 11bp nucleotides (869-879), and introducing a single nucleotide A > G conversion at position 882 (Supplementary Figure 1A) and WT mothers. Since 6302 is a single copy mutant pig, the progeny will contain both MSTN^+/−^ and WT pigs. Ear samples were collected one month after birth for genotyping. The WT DNA sequences showed single peaks throughout the *MSTN* gene, while the mutant DNA sequences showed multiple, overlapping, divergent base calls starting after position 868 (see Supplementary Figure 1B).

### Microbiome of Intestinal Contents From WT and MSTN^+/−^ Large White Pigs

**FIGURE 1:**
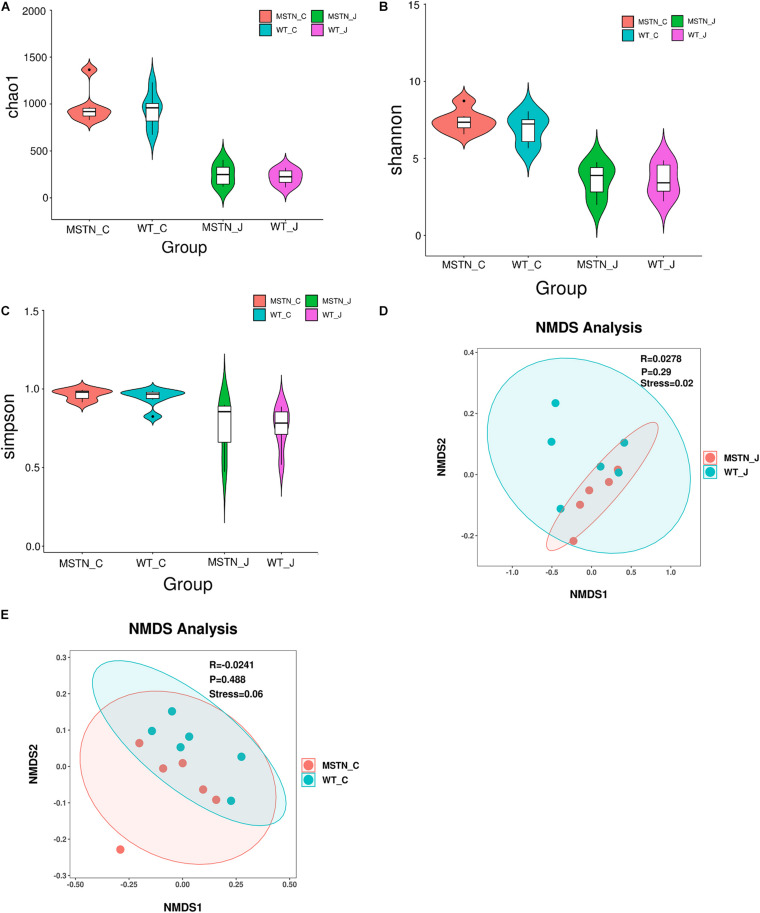
Gut microbiome diversity and structure analysis. **(A–C)** Violin plots showing chao1 **(A)**, shannon **(B)**, and simpson **(C)** of indices in both WT and MSTN^+/−^ jejunum and cecum samples; **(D,E)** Non-metric multi-dimensional scaling (NMDS) ordination plot derived from weighted pairwise UniFrac distances in both WT and MSTN^+/−^ jejunum **(D)**, and cecum **(E)** samples. Stress values for ordination plot were <0.1, which indicates the accuracy of data representation in a two-dimensional space.

We used a total of 24 samples consisting of the jejunum and the cecum content from WT and MSTN groups to study gut microbiota diversity through 16S rDNA (V3-V4 region) high-throughput sequencing. We evaluated differences in bacterial diversity between the MSNT and WT groups in both jejunum and cecum samples aligning these sequences and estimating alpha and beta diversity indices. The Chao1 index, Shannon and Simpson indexes, indicators of microbial richness were no statistically significant differences between groups (Figures 1A–C). β-diversity was assessed by Weighted UniFrac distance-based Non-metric Multidimensional Scaling (NMDS), which is a powerful ordination method that allows to uncover non-linear relationships between samples. The NMDS-based maps of the jejunum showed distinct differences between wild-type (WT_J) and MSTN -edited (MSTN_J) pigs (stress value = 0.02, Figure 1D). Similar distinct differences were observed when comparing cecum sample (stress value = 0.06, Figure 1F). Our results further showed that MSTN editing had no significant effect on the bacterial abundance and diversity of despite the aforementioned changes in the gut microbiota composition in the jejunum and the cecum of MSTN pigs.

**FIGURE 2:**
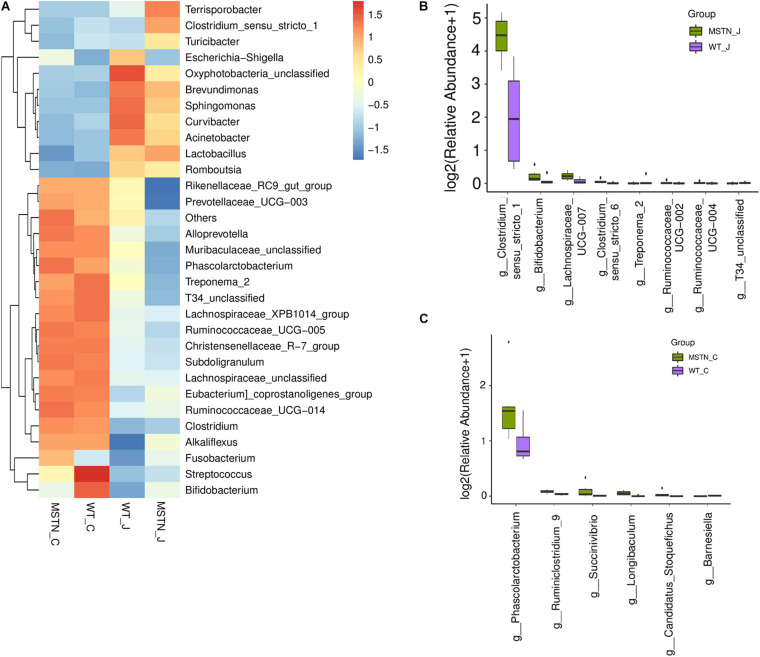
Generic differences between WT and MSTN groups. **(A)** Heat map of relative abundance of 30 genera. **(B)** Significantly different genera between WT and MSTN cecum samples. **(C)** Significantly different genera between WT and MSTN jejunum samples.

Next, we assessed the overall bacterial community compositions using heatmaps displaying the abundance of taxa at the phylum (Supplementary Figure 2A) and genus (Figure 2A) levels. At the phylum level, we found an downregulated of *Spirochaetes* in the jejunum of MSTN^+/−^ pigs (Supplementary Figure 2B). At the genus level, *Lactobacillus* (44.99%), *Clostridium_sensu_stricto* (22.00%), *Terrisporobacter* (14.11%) and *Turicibacter* (3.08%) were the predominant genera in the MSTN jejunum; while *Lactobacillus* (39.63%), *Escherichia-Shigella* (14.87%), *Brevundimonas* (8.20%) and *Clostridium_sensu_stricto* (4.85%) were the most abundant in the WT jejunum. Wilcoxon Rank Sum Test was performed to compare the relative abundance of species in the two groups. *Clostridium_sensu_stricto_1* (*p* = 0.0065), *Bifidobacterium* (*p* = 0.0374), *Lachnospiraceae_UCG-007* (*p* = 0.0374), *Clostridium_sensu_stricto_6* (*p* = 0.0061), *Ruminococcaceae_UCG-002* (*p* = 0. 0.0495), and *Ruminococcaceae_UCG-004* (*p* = 0. 0.0495) were significantly upregulated in MSTN_J;while *Treponema_2* (*p* = 0.021) and *T34_unclassified* (*p* = 0.0028) were significantly downregulated (Figure 2B).

In cecum samples, *Lactobacillus* (15.59%), *Ruminococcaceae_UCG-005* (10.13%), *Escherichia-Shigella* (4.40%) and *Clostridium_sensu_stricto_1* (4.14%) were the most abundant genera in the MSTN_C group; while *Lactobacillus* (18.55%), *Streptococcus* (11.03%), *Ruminococcaceae_UCG-005* (8.26%) and *Clostridium_sensu_stricto_1* (5.22%)%) were the most frequent in the WT_C group. A comparison between these groups showed a significant upregulated of *Phascolarctobacterium* (*p* = 0.0374), *Ruminiclostridium_9* (*p* = 0.0163), *Succinivibrio* (*p* = 0.0278), *Longibaculum* (*p* = 0.0033), and *Candidatus_Stoquefichus* (*p* = 0.0463) in the MSTN group. On the contrary, *Barnesiella* (*p* = 0.0201) was significantly downregulated (Figure 2C).

**FIGURE 3:**
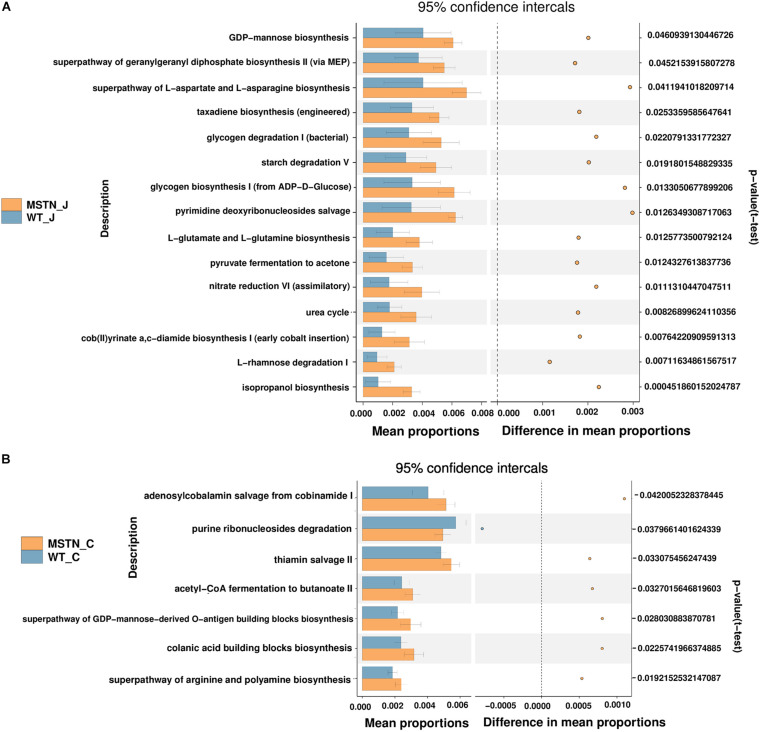
Comparison of PICRUSt-predicted functional pathways in the jejunum and the cecum between WT and MSTN^+/−^ groups. **(A,B)** The extended error bars method in STAMP showing relative difference pathway in comparisons involving one hypervariable dataset and other datasets using Welch’s t-test (two sided).

We predicted the functional potential of bacterial communities in WT and MSTN samples using the PICRUSt2 software (Langille et al., 2013). KEGG pathway analyses unveiled a total of 15 significant pathways were classified in MSTN jejunum samples (Figure 3A), in particular L-rhamnose degradation I (the most significant). Moreover, we were able to find 7 significantly different KEGG pathways in MSTN cecum samples, including acetyl-CoA fermentation to butanoate II, adenosylcobalamin salvage from cobinamide I, colanic acid building blocks biosynthesis, purine ribonucleosides degradation, superpathway of arginine and polyamine biosynthesis, superpathway of GDP-mannose-derived O-antigen building blocks biosynthesis and thiamin salvage II (Figure 3B).

### Non-targeted Metabolomics of Fecal Samples Form WT and MSTN^+/−^ Large White Pigs

**FIGURE 4:**
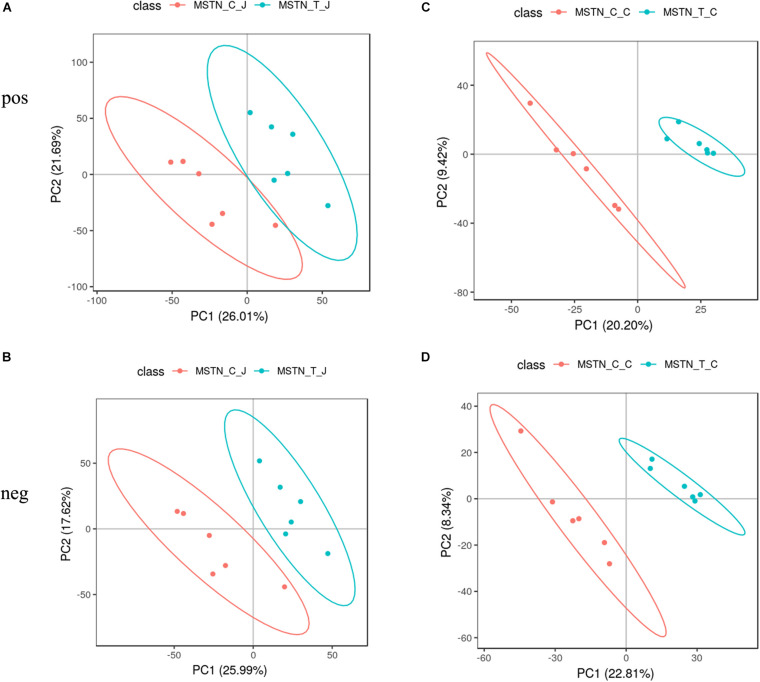
PLS-DA plot based on fecal metabolites. **(A–D)** PLS-DA plot of fecal metabolites detected in positive and negative mode between MSTN and WT pigs in the jejunum **(A,B)** and cecum **(C,D)**.

To evaluate the metabolic changes occurring as a response to *MSTN* gene editing, we analyzed a total of 24 intestinal contents from the jejunum and cecum of WT and MSTN^+/−^ Large White pigs (n = 6 for each group) by LC-MS-based untargeted metabolomics. This analysis allowed us to identify a total of 805 metabolites from experiments including both positive and negative ion modes. We then performed a partial least-squares-discriminant analysis (PLS-DA) to identify the altered metabolites in MSTN-edited Large White pigs. The PLS-DA model revealed significant differences between WT and MSTN^+/−^ samples in the same intestinal tract (Figures 4A–D). Specifically, our PLS-DA model revealed metabolic profile differences between WT_J and MSTN_J (Figures 3A,B), WT_I and MSTN_I (Figures 4A,B), and WT_C and MSTN_C (Figures 4C,D), suggesting that *MSTN* gene editing leads to significant biochemical changes in the gut. The R2 and Q2 values of all mathematical models used are summarized in Supplementary Table 3.

**FIGURE 5:**
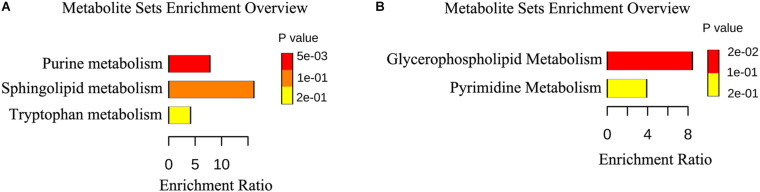
Metabolic pathway enrichment analysis. **(A,B)** Overview of metabolites that were changed in the jejunum **(A)** and the cecum **(B)** of MSTN pigs compared to WT.

We identified 15 differently enriched metabolites between MSTN_J and WT_J based on VIP values and relative abundance, of which 5 were up-regulated and 10 were down-regulated in MSTN_J samples (Table 1). These metabolites were mainly involved in purine, sphingolipid and tryptophan metabolism (Figure 5A).

**TABLE 1:** Fold changes (FC) for LC-MS-based significant different metabolomics datasets for the MSTN_J and WT_J groups.

Metabolites	FC (MSTN_J/WT_J)	*p*-value	VIP	Regulated	HMD	KEGG	Class
Acylcarnitine 20:3	0.4430	0.0278	1.7653	down	-	C02301	Fatty Acyls
Indoleacetic acid	0.3444	0.0167	2.6622	down	HMDB0000197	C00954	Indoles and derivatives
Stearidonic acid ethyl ester	0.4983	0.0108	1.4994	down	NA	NA	Fatty Acyls
trans-13-Octadecenoic acid	0.4872	0.0489	1.9726	down	NA	NA	Fatty Acyls
Trp-Ile	0.1674	0.0031	3.3156	down	HMDB0029086	NA	Carboxylic acids and derivatives
4-Pyrimidinamine, 5-cyclopropyl-2-[1-[(2-fluorophenyl)methyl]-1H-pyrazolo[3,4-b]pyridin-3-yl]-	2.0251	0.0358	1.7076	up	NA	NA	-
Acylcarnitine 18:2	3.1434	0.0440	2.1855	up	HMDB06461	C02301	Fatty Acyls
Cholesta-4,6-dien-3-one	3.7609	0.0432	2.3096	up	HMDB0002394	NA	Sterol Lipids
D-erythro-N-stearoylsphingosine	2.2831	0.0237	1.2371	up	HMDB0000252	C00319	Organonitrogen compounds
D-erythro-Sphinganine	3.5663	0.0175	2.2467	up	HMDB0000269	C00836	Organonitrogen compounds
Guanine	3.5499	0.0103	2.3976	up	HMDB0000132	C00242	Imidazopyrimidines
Guanosine	5.4285	0.0353	2.1855	up	HMDB0000133	C00387	Purine nucleosides
Heptadecasphinganine	2.0789	0.0310	1.1752	up	NA	NA	-
Inosine	4.7907	0.0029	2.7243	up	HMDB0000195	C00294	Purine nucleosides
L-Cysteine S-sulfate	3.7617	0.0123	2.0186	up	HMDB0000731	C05824	Carboxylic acids and derivatives
							

Analogously, we found 22 differently enriched metabolites between the MSTN_C and WT_C samples, of which 7 were up-regulated and 14 were down-regulated in the former. Half of these metabolites were classified as lipids including fatty acyls, sterol lipids, prenol lipids and glycerophospholipids (Table 2). Functional analysis of these metabolites revealed that they were mainly involved in glycerophospholipid and pyrimidine metabolism (Figure 5B).

**TABLE 2:** Fold changes (FC) for LC-MS-based significant different metabolomics datasets for the MSTN_C and WT_C groups.

Metabolite	FC (MSTN_C/WT_C)	*p*-value	wilcox.test_*p*.value	VIP	Regulated	HMD	KEGG	MS2class
13-HODE	0.4684	0.0473	0.0650	1.7067	down	HMDB0061708	null	Fatty Acyls
1b,3a,12a-Trihydroxy-5b-cholanoic acid	0.3959	0.0383	0.0411	1.6173	down	HMDB0000326	null	Sterol Lipids
(3’RS,3’SR)-Astaxanthin	0.3865	0.0369	0.0411	3.0134	down	HMDB0039128	null	Prenol lipids
2-Indolinone	0.4540	0.0287	0.0411	2.0002	down	NA	NA	-
Benzothiazole	0.4211	0.0372	0.0931	2.2433	down	HMDB0032930		Benzothiazoles
3-Methylguanine	0.4256	0.0211	0.0152	1.8673	down	HMDB0001566	C02230	Imidazopyrimidines
N-Acetyl-L-phenylalanine	0.3614	0.0170	0.0411	2.1251	down	HMDB0000512	C03519	Carboxylic acids and derivatives
2-(4-Methoxyphenyl)naphthalic anhydride	0.4777	0.0149	0.0152	1.8517	down	HMDB0032909	null	Naphthalenes
12,13-Dihydroxy-9Z-octadecenoic acid	0.4841	0.0167	0.0260	2.2591	down	HMDB0004705	C14829	Fatty Acyls
2-Linoleoylglycerol	0.4459	0.0052	0.0043	1.9289	down	HMDB0011538	NA	Fatty Acyls
Acylcarnitine 17:2	0.4226	0.0215	0.0152	2.8360	down	-	C02301	Fatty Acyls
Peimine	0.4183	0.0214	0.0152	2.8521	down	NA	C10830	-
Acylcarnitine 19:4	0.4927	0.0027	0.0043	1.2353	down	-	C02301	Fatty Acyls
Acylcarnitine 20:3	0.3083	0.0126	0.0260	2.3226	down	-	C02301	Fatty Acyls
Ganoderiol F	0.4074	0.0084	0.0087	1.8565	down	HMDB0038707	null	Prenol lipids
Termitomycamide E	4.8589	0.0118	0.0152	1.6471	up	NA	NA	-
LysoPG 18:0; LysoPG 18:0	4.3783	0.0140	0.0260	2.2877	up	-	C05980	Glycerophospholipids
2-Piperidinone	2.6932	0.0143	0.0152	1.9661	up	HMDB0011749	null	Piperidines
Thymidine	4.4249	0.0108	0.0260	2.6997	up	HMDB0000273	C00214	Pyrimidine nucleosides
trans-13-Octadecenoic acid	2.1795	0.0468	0.0931	2.6609	up	NA	NA	Fatty Acyls
Erucamide	2.6252	0.0045	0.0043	2.9174	up	NA	NA	-
LysoPE 18:0	2.2495	0.0095	0.0260	1.1495	up	HMDB11129;HMDB11130	C04438	Glycerophospholipids

### Correlation of Gut Microbiota With Fecal Metabolic Phenotype

**FIGURE 6:**
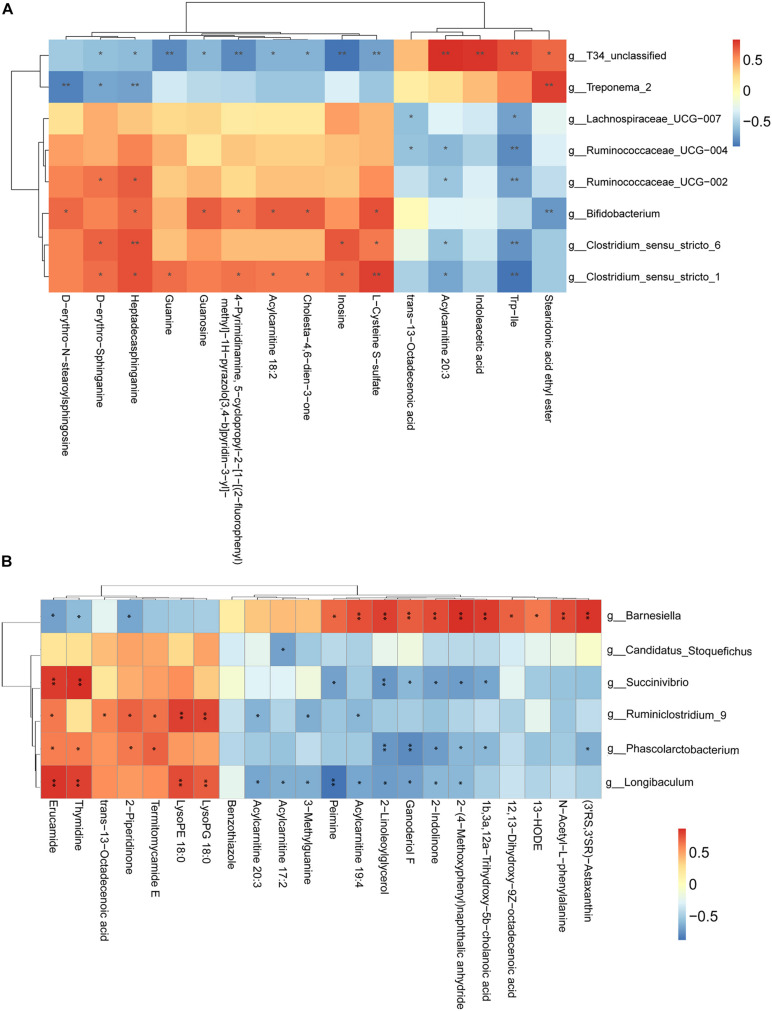
Correlation analysis between perturbed gut microbes and altered metabolites. **(A,B)** Pearson’s correlation analysis between perturbed genera and metabolite concentrations in the jejunum **(A)** and the cecum **(B)**. Asterisks indicate significant correlations between MSTN and WT pigs. Cells are according to Spearman’s correlation coefficient between the significantly altered genera and metabolites; significantly positive correlation (*P* < 0.05), and significantly negative (*P* < 0.05) correlations are shown in red and blue, respectively. ^∗^*P* < 0.05, ^∗∗^*P* < 0.01.

We have also conducted correlation analyses between the changed relative abundance of gut bacterial taxa and altered fecal metabolites, which were visualized in a heatmap (Figure 6). In general, we found an agreement between the observed taxa enrichment and metabolites presence in the jejunum and samples. Network regulation analysis results of altered metabolites and genera in the jejunum and the cecum are showed in Supplementary Figure 3.

In summary, we demonstrated that MSTN gene editing changed the structure and composition of the gut microbiome and altered fecal metabolic configuration in the jejunum and the cecum of MSTN^+/−^ Large White pigs. Moreover, we showed that a strong correlation between the significant differential metabolites and significant changed genera in jejunum and cecum. Non-targeted metabolomics identified a number of metabolites that affected gene editing, possibly, at least in part, due to ecological changes in the microbiota.

## Discussion

In this study, we aimed to determine the effects of *MSTN* gene editing on the fecal microbiome and metabolome. Accordingly, we employed an integrated approach consisting of 16S rDNA sequencing and LC-MS-based untargeted metabolomics to explore the phenotypical changes in gut microbiota and fecal metabolic composition in the jejunal and cecal of *MSTN*-edited Large White pigs.

Despite the overall similarities in the composition of the fecal microbiome of at a phylum level, Wilcoxon Rank Sum Test analyses revealed differences in the microbial profiles of *MSTN*-edited and WT groups, which indicate that *MSTN* gene editing is associated with shifts in the relative abundance of individual gut bacterial species. The alpha diversity of intestinal microbiota in the jejunum and the cecum was not observed between the two groups. However, we found that the beta diversity of MSTN intestinal contents was altered. Besides, several genus microbes in both the jejunum and cecum were seen to up/down regulated MSTN^+/−^ Large White pigs. These findings somehow indicated that the *MSTN* gene editing not only changes the proportion of lean meat in Large White pigs (data no showed in this article), and also the composition of microorganisms in the gut. The result is inconsistent with a previous research using rectal feces of *MSTN* double-allele knockout Meishan pigs (Cui et al., 2019). The differences may arise from analyzing distinct pig species, different genotypes and from employing different methodological approaches for data analysis (e.g., QIIME 2 does not performed clustering based on sequence similarity but instead revises the sequencing errors of amplitors through dereplication, chimeric filtering and other methods to improve accuracy).

The alpha diversity of the microbiome, to some degree, is a health indicator of the host. In mice, social stress significantly changes microbial population (Bailey et al., 2010) and reduces the alpha diversity of gut microbiome (Bailey et al., 2011). Stress-induced cage rearing reduces gut microbial diversity in chickens as compared to free-range rearing (Chen et al., 2019), while heat stress results in decreased gut microbial diversity in Holstein dairy cows (Chen et al., 2018). Here, no changes in the intestinal microbial alpha diversity of MSTN gene editing pigs likely indicates no change in the health status of MSTN pigs.

The gut microbiota comprises numerous microorganisms, and changes in the gut microbial composition are influenced by both the host genotype and environmental factors (Neish, 2009; David et al., 2014). Results showed that 2 genotypes, GG and TT, in the rs16775833 within the DMRT1 gene can modulate the microbial community structure and are associated with the body weight of birds (Ji et al., 2020). The host’s genetic background, including the different isoforms of the apolipoprotein E (APOE) gene, can exert an influence over microbiota composition (Guardia-Escote et al., 2019). All pigs were sampled under the same feeding conditions throughout the experiment, but the gut microbial composition of MSTN pig jejunum and cecum microbes were altered, most likely due to changes in the *MSTN* gene. The jejunum is specialized in absorbing small nutrient particles via the enterocytes, and the mammal intestinal microbiome plays a key role in converting food into useable nutrients for the host. In the present study, concordant changes in both metabolite presence and microorganism composition in the jejunum were obtained. *Clostridium_sensu_stricto_1*, a genus belonging to butyrate-producing *Clostridia* bacteria, increased significantly in the MSTN group. Butyrate has a significant part in the development of intestinal epithelial cells and is essential for the energy supply of the host as the final unabsorbed polysaccharide metabolite in the fermented food of the intestinal microbiota (Pryde et al., 2002). Hence, our observations suggest an upregulation in the production of butyrate in the MSTN group that subsequently enhances energy metabolism. *Bifidobacterium* is associated with health promoting outcomes due to the production of bacteriocins (Martinez et al., 2013), and might contribute to improved metabolic end-products (e.g., acetate and lactate) (Alcon-Giner et al., 2020). Accordingly, the upregulated of *Bifidobacterium* in the MSTN group indicate that the production of acetate, lactate and bacteriocins is likely upregulated in MSTN group, which promotes energy metabolism and health. Interestingly, the main function of this genus is to ferment sugars into volatile fatty acids, CO_2_ and H_2_ (Berkessa et al., 2020), whereby an enrichment of *Clostridium_sensu_stricto_6* in the MSTN group suggest that MSTN pigs might have enhanced sugar metabolism. The *Ruminococcaceae_UCG-002* is a hydrolytic-acidogenic bacteria, that is known for its role in the organic compounds during cation exchange resin-induced hydrolysis of waste activated sludge. Some of these compounds include tryptophan-like and tyrosine-like proteins, amino acids, aliphatic, and metabolic intermediates (Xiao et al., 2020). The *Ruminococcaceae_UCG-004* has been associated with intestinal permeability indices (Gao et al., 2019). The significant overrepresentation of these two genera in MSTN_J suggested that the absorptive capacity of the intestinal wall may be stronger, and that digestion of substances in the intestinal tract is more efficient compared to wild-type. The increase of *Phascolarctobacterium* in the cecum of *MSTN*-edited pigs with low fat content is consistent with previous results showing a negative correlation between *Phascolarctobacterium* and the percentage of body fat (Naderpoor et al., 2019). A decline in the abundance of *Phascolarctobacterium* has been associated with inflammatory diseases, including colorectal cancer, whereby this bacteria might reduce colonic inflammation, enhance protection of the colonic mucosa and provide nutrition to the colon cells. It is possible that similar processes occur in the cecum as a result of an increase in *Phascolarctobacterium* in MSTN samples.

The main function of the cecum is to absorb the remaining fluids and salts after intestinal digestion and absorption are completed, and to mix these contents with mucus, a lubricating substance. We identified several overrepresented genera in this intestinal segment. Among these is *Ruminiclostridium_*9, a bacterial group which has been previously connected to obesity and other metabolic disorders (Wang et al., 2019), although these results are regarded with caution. While some studies have proposed that obese mice have a higher abundance of *Ruminiclostridium_9* in their fecal microbiota than the leaner counterparts (Hou et al., 2019; Luo et al., 2019; Wang et al., 2020), others have shown that *Ruminiclostridium_9* is instead negatively correlated with obesity (Zhao et al., 2017; Zhu et al., 2018; Hu et al., 2019). Another example of a MSTN-edited upregulated group in the cecum is *Succinivibrio*, a bacteria that has high potential for fiber degradation (Hippe et al., 1999) and thus can improve metabolism. Enrichment of *Succinivibrio* in the cecum might also provide the host with additional nutrients.

In addition to alterations in the fecal composition of microbiota in MSTN-edited pigs, we implemented, for the first time, a comprehensive analysis of the fecal metabolome of the intestinal MSTN-edited samples. The disruption of the gut microbiota homeostasis subsequently affects intestinal metabolism (Alou et al., 2016; Lippert et al., 2017), whereby we envisioned that *MSTN* gene editing-associated changes in microbial composition might be followed by changes in the relative abundance of different metabolites in fecal samples. We were able to support this hypothesis by demonstrating differentially detected metabolites in both the jejunum and the cecum. We found differential metabolites in the contents of the ileum and cecum, and a lot of these were accounted for by fatty acids, sterol lipids and glycerophospholipids (Fahy et al., 2009). Functional analysis showed they were involved in metabolism of sphingolipid (jejunum) and glycerophospholipid (cecum). Both sphingolipid and glycerophospholipid metabolism belong to the lipid metabolism pathway. A recent study reported the protective roles of Huangjinya green tea extract against obesity, liver steatosis and insulin resistance in high-fat diet-fed mice, highlighting its favorable modulation on fecal metabolites of amino acids, sphingolipids, and bile acids *in vivo*(Li et al., 2020). Another study found that oral hydroxysafflor yellow can reduce obesity in mice and the changed metabolites by serum metabolomics analysis were mainly linked with the pathways of glycerophospholipid and sphingolipid metabolism (Liu et al., 2018). These studies suggest that the resistance to obesity in animals may cause changes in the lipid metabolic pathways. The ratio of fat of MSTN-edited pigs was 100% lower than that of WT ([| heterozygote value–original value of WT|]/original value of WT). Correlation analysis allowed us to identify several bacterial genera potentially implicated in the host metabolic. For instance, in the jejunum, *T34_unclassified*, *Bifidobacterium* and *Clostridium_sensu_stricto_1* were positively correlated with the majority of metabolites that were changed in the MSTN pigs. In the cecum, *Barnesiella* abundance was negatively correlated with 3 metabolites and positively correlated with 11 metabolites, while *Longibaculum* was positively and negatively correlated with 4 metabolites and 9 metabolites, respectively. The strongly correlation between the differentially detected metabolites with flora composition Hence, it is possible that these changes are consistent with phenotype and are caused by alterations in the microflora.

## Conclusion

In conclusion, our study showed that *MSTN* gene editing alters the composition of metabolites and microbial communities in the jejunum and the cecum. Our results demonstrate that the different microbiome composition in the jejunal and cecal may provide more useable nutrients for the host of *MSTN*-edited pigs and influence the composition of metabolites.

## Data Availability Statement

The datasets presented in this study can be found in online repositories. The names of the repository/repositories and accession number(s) can be found below: https://www.ncbi.nlm.nih.gov/sra/PRJNA687099.

## Ethics Statement

The animal study was reviewed and approved by Animal Welfare and Research Ethics Committee at the Institute of Animal Sciences, Chinese Academy of Agricultural Sciences (CAAS).

## Author Contributions

KL and HL designed and managed the project. YP and CC analyzed the data and performed all animal works and collected biological samples. YP wrote the manuscript. HL, YY, ZF, BL, HL, and KL revised the manuscript. All authors approved the final version of the manuscript.

## Conflict of Interest

The authors declare that the research was conducted in the absence of any commercial or financial relationships that could be construed as a potential conflict of interest.

## Supplementary Material

Supplementary Figure 1 Genotype identification results. **(A)** The mutation domain’s specific sequence of MSTN mutant large white pigs. **(B)** Genome sequencing results of heterozygote MSTN mutant and wild-type large white pigs.

Supplementary Figure 2 Phylum differences between WT and MSTN groups. **(A)** Heat map showing the relative abundance of Phyla. **(B)** Significantly different genera in WT and MSTN jejunum samples.

Supplementary Figure 3 Altered metabolites and genera network regulation analysis results. **(A,B)** Network regulation analysis results of altered metabolites and genera in the jejunum **(A)** and the cecum **(B)**. The different nodes in the figure represent different bacterial communities or metabolites. Bacterial communities are represented by the round shapes while metabolites are represented by triangles. Solid and dashed lines represent the positive and negative correlations between the flora and the metabolites, respectively.

Supplementary Table 1 Information about of pigs in this study.

Supplementary Table 2 Composition and nutrient levels of the diets (%, as-fed basis).

Supplementary Table 3 The R2 and Q2 values for all mathematical models.

## References

[B1] Alcon-Giner C. Dalby M. J. Caim S. Ketskemety J. Shaw A. Sim K. (2020). Microbiota Supplementation with Bifidobacterium and Lactobacillus Modifies the Preterm Infant Gut Microbiota and Metabolome: An Observational Study. *Cell Rep. Med.* 1:100077. 10.1016/j.xcrm.2020.100077 32904427 PMC7453906

[B2] Alou M. T. Lagier J. C. Raoult D. (2016). Diet influence on the gut microbiota and dysbiosis related to nutritional disorders. *Hum. Microbiome J.* 1 3–11. 10.1016/j.humic.2016.09.001

[B3] Bailey M. T. Dowd S. E. Galley J. D. Hufnagle A. R. Allen R. G. Lyte M. (2011). Exposure to a Social Stressor Alters the Structure of the Intestinal Microbiota: Implications for Stressor-Induced Immunomodulation. *Brain Behav. Immun.* 25 397–407. 10.1016/j.bbi.2010.10.023 21040780 PMC3039072

[B4] Bailey M. T. Dowd S. E. Parry N. M. A. Galley J. D. Schauer D. B. Lyte M. (2010). Stressor Exposure Disrupts Commensal Microbial Populations in the Intestines and Leads to Increased Colonization by Citrobacter rodentium. *Infect. Immun.* 78 1509–1519. 10.1128/iai.00862-09 20145094 PMC2849416

[B5] Berkessa Y. W. Yan B. Li T. Jegatheesan V. Zhang Y. (2020). Treatment of anthraquinone dye textile wastewater using anaerobic dynamic membrane bioreactor: Performance and microbial dynamics. *Chemosphere* 238:124539. 10.1016/j.chemosphere.2019.124539 31470310

[B6] Chen S. Wang J. Peng D. Li G. Chen J. Gu X. (2018). Exposure to heat-stress environment affects the physiology, circulation levels of cytokines, and microbiome in dairy cows. *Sci. Rep.* 8:14606. 10.1038/s41598-018-32886-1 PMC6168502 30279428

[B7] Chen S. Xiang H. Zhang H. Zhu X. Wang D. Wang J. (2019). Rearing system causes changes of behavior, microbiome, and gene expression of chickens. *Poultry Sci.* 98 3365–3376. 10.3382/ps/pez140 30916350

[B8] Cui W. T. Xiao G. J. Jiang S. W. Qian L. L. Cai C. B. Li B. (2019). Effect of ZFN-edited myostatin loss-of-function mutation on gut microbiota in Meishan pigs. *PLoS One* 14:e0210619. 10.1371/journal.pone.0210619 30645618 PMC6333347

[B9] David L. A. Maurice C. F. Carmody R. N. Gootenberg D. B. Button J. E. Wolfe B. E. (2014). Diet rapidly and reproducibly alters the human gut microbiome. *Nature* 505 559–563. 10.1038/nature12820 24336217 PMC3957428

[B10] Fahy E. Subramaniam S. Murphy R. C. Nishijima M. Dennis E. A. (2009). Update of the LIPID MAPS comprehensive classification system for lipids. *J. Lipid Res.* 50 Suppl(Suppl.), S9–S14. 19098281 10.1194/jlr.R800095-JLR200 PMC2674711

[B11] Gao Z. Wu H. Zhang K. Hossen I. Cao Y. (2019). Protective effects of grape seed procyanidin extract on intestinal barrier dysfunction induced by a long-term high-fat diet. *J. Funct. Foods* 64:103663. 10.1016/j.jff.2019.103663

[B12] Guardia-Escote L. Basaure P. Biosca-Brull J. Cabré M. Colomina M. T. (2019). APOE genotype and postnatal chlorpyrifos exposure modulate gut microbiota and cerebral short-chain fatty acids in preweaning mice. *Food Chem. Toxicol.* 135:110872. 10.1016/j.fct.2019.110872 31622728

[B13] Guo T. Jou W. Chanturiya T. Portas J. Gavrilova O. McPherron A. C. (2009). Myostatin inhibition in muscle, but not adipose tissue, decreases fat mass and improves insulin sensitivity. *PLoS One* 4:e4937. 10.1371/journal.pone.0004937 19295913 PMC2654157

[B14] Hippe H. Hagelstein A. Kramer I. Swiderski J. Stackebrandt E. (1999). Phylogenetic analysis of Formivibrio citricus, Propionivibrio dicarboxylicus, *Anaerobiospirillum* thomasii, Succinimonas amylolytica and Succinivibrio dextrinosolvens and proposal of Succinivibrionaceae fam. nov. *Int. J. Syst. Bacteriol.* 49(Pt 2), 779–782. 10.1099/00207713-49-2-779 10319502

[B15] Hou D. Zhao Q. Yousaf L. Khan J. Shen Q. (2019). Consumption of mung bean (Vigna radiata L.) attenuates obesity, ameliorates lipid metabolic disorders and modifies the gut microbiota composition in mice fed a high-fat diet. *J. Funct. Foods* 64:103687. 10.1016/j.jff.2019.103687

[B16] Hu S. Xu Y. Gao X. Li S. Jiang W. Liu Y. (2019). Long-Chain Bases from Sea Cucumber Alleviate Obesity by Modulating Gut Microbiota. *Mar. Drugs* 17:17080455. 10.3390/md17080455 31374958 PMC6723202

[B17] Ji J. Xu Y. Luo C. He Y. Xu X. Yan X. (2020). Effects of the DMRT1 genotype on the body weight and gut microbiota in the broiler chicken. *Poultry Sci.* 99 4044–4051. 10.1016/j.psj.2020.03.055 32731992 PMC7597928

[B18] Langille M. G. I. Zaneveld J. Caporaso J. G. McDonald D. Knights D. Reyes J. A. (2013). Predictive functional profiling of microbial communities using 16S rRNA marker gene sequences. *Nat. Biotechnol.* 31, 814–821. 10.1038/nbt.2676 23975157 PMC3819121

[B19] Li M. Xu J. Zhang Y. Chu S. Sun S. Huo Y. (2020). Comparative analysis of fecal metabolite profiles in HFD-induced obese mice after oral administration of huangjinya green tea extract. *Food Chemic. Toxicol.* 145:111744. 10.1016/j.fct.2020.111744 32918987

[B20] Li Y. Fang J. Qi X. Lin M. Zhong Y. Sun L. (2018). Combined Analysis of the Fruit Metabolome and Transcriptome Reveals Candidate Genes Involved in Flavonoid Biosynthesis in Actinidia arguta. *Int. J. Mol. Sci.* 19:19051471. 10.3390/ijms19051471 29762529 PMC5983832

[B21] Lippert K. Kedenko L. Antonielli L. Kedenko I. Gemeier C. Leitner M. (2017). Gut microbiota dysbiosis associated with glucose metabolism disorders and the metabolic syndrome in older adults. *Benefic. Microbes* 8 1–12. 10.3920/BM2016.0184 28701081

[B22] Liu J. Yue S. Yang Z. Feng W. Meng X. Wang A. (2018). Oral hydroxysafflor yellow A reduces obesity in mice by modulating the gut microbiota and serum metabolism. *Pharmacol. Res.* 134 40–50. 10.1016/j.phrs.2018.05.012 29787870

[B23] Lu D. Tiezzi F. Schillebeeckx C. McNulty N. P. Schwab C. Shull C. (2018). Host contributes to longitudinal diversity of fecal microbiota in swine selected for lean growth. *Microbiome* 6:4. 10.1186/s40168-017-0384-1 29301569 PMC5755158

[B24] Luo Q. Cheng D. Huang C. Li Y. Lao C. Xia Y. (2019). Improvement of Colonic Immune Function with Soy Isoflavones in High-Fat Diet-Induced Obese Rats. *Molecules* 24:1139. 10.3390/molecules24061139 30909396 PMC6470843

[B25] Mach N. Berri M. Estellé J. Levenez F. Lemonnier G. Denis C. (2015). Early-life establishment of the swine gut microbiome and impact on host phenotypes. *Environ. Microbiol. Rep.* 7 554–569. 10.1111/1758-2229.12285 25727666

[B26] Maltecca C. Bergamaschi M. (2020). The interaction between microbiome and pig efficiency: A review. *J. Anim. Breed Genet.* 137 4–13. 10.1111/jbg.12443 31576623

[B27] Martinez F. A. Balciunas E. M. Converti A. Cotter P. D. de Souza Oliveira R. P. (2013). Bacteriocin production by Bifidobacterium spp. *Rev. Biotechnol. Adv.* 31 482–488. 10.1016/j.biotechadv.2013.01.010 23384878

[B28] Matika O. Robledo D. (2019). Balancing selection at a premature stop mutation in the myostatin gene underlies a recessive leg weakness syndrome in pigs. *PLoS Genet.* 15:e1007759. 10.1371/journal.pgen.1007759 30699111 PMC6370237

[B29] McPherron A. C. Lawler A. M. Lee S. J. (1997). Regulation of skeletal muscle mass in mice by a new TGF-beta superfamily member. *Nature* 387 83–90. 10.1038/387083a0 9139826

[B30] Naderpoor N. Mousa A. Gomez-Arango L. F. Barrett H. L. Nitert M. D. Courten B. D. (2019). Faecal Microbiota Are Related to Insulin Sensitivity and Secretion in Overweight or Obese Adults. *J. Clin. Med.* 8:452. 10.3390/jcm8040452 30987356 PMC6518043

[B31] Neish A. S. (2009). Microbes in gastrointestinal health and disease. *Gastroenterology* 136 65–80. 10.1053/j.gastro.2008.10.080 19026645 PMC2892787

[B32] Park S. J. Kim J. Lee J. S. Rhee S. K. Kim H. (2014). Characterization of the fecal microbiome in different swine groups by high-throughput sequencing. *Anaerobe* 28 157–162. 10.1016/j.anaerobe.2014.06.002 24954845

[B33] Pryde S. E. Duncan S. H. Hold G. L. Stewart C. S. Flint H. J. (2002). The microbiology of butyrate formation in the human colon. *FEMS Microbiol. Lett.* 217 133–139. 10.1111/j.1574-6968.2002.tb11467.x 12480096

[B34] Qian L. Tang M. Yang J. Wang Q. Cai C. Jiang S. (2015). Targeted mutations in myostatin by zinc-finger nucleases result in double-muscled phenotype in Meishan pigs. *Rep* 5:14435. 10.1038/srep14435 PMC4585837 26400270

[B35] Ramayo-Caldas Y. Mach N. Lepage P. Levenez F. Denis C. Lemonnier G. (2016). Phylogenetic network analysis applied to pig gut microbiota identifies an ecosystem structure linked with growth traits. *ISME J.* 10 2973–2977. 10.1038/ismej.2016.77 27177190 PMC5148198

[B36] Rao S. Fujimura T. Matsunari H. Sakuma T. Nakano K. Watanabe M. (2016). Efficient modification of the myostatin gene in porcine somatic cells and generation of knockout piglets. *Mol. Reprod. Dev.* 83 61–70. 10.1002/mrd.22591 26488621

[B37] Wang K. Ouyang H. Xie Z. Yao C. Guo N. Li M. (2015). Efficient Generation of Myostatin Mutations in Pigs Using the CRISPR/Cas9 System. *Sci. Rep.* 5:16623. 10.1038/srep16623 26564781 PMC4643223

[B38] Wang K. Tang X. Xie Z. Zou X. Li M. Yuan H. (2017). CRISPR/Cas9-mediated knockout of myostatin in Chinese indigenous Erhualian pigs. *Transgenic Res.* 26 799–805. 10.1007/s11248-017-0044-z 28993973

[B39] Wang P. Gao J. Ke W. Wang J. Hu X. (2020). Resveratrol reduces obesity in high-fat diet-fed mice via modulating the structure and metabolic function of the gut microbiota. *Free Radic. Biol. Med.* 156 83–98. 10.1016/j.freeradbiomed.2020.04.013 32305646

[B40] Wang R. Li S. Jin L. Zhang W. Wu C. (2019). Four-week administration of nicotinemoderately impacts blood metabolic profile and gut microbiota in a diet-dependent manner. *Biomed. Pharmacother.* 115:108945. 10.1016/j.biopha.2019.108945 31100541

[B41] Xiao K. Abbt-Braun G. Horn H. (2020). Changes in the characteristics of dissolved organic matter during sludge treatment: A critical review. *Water Res.* 187:116441. 10.1016/j.watres.2020.116441 33022515

[B42] Xiao L. Estellé J. Kiilerich P. Ramayo-Caldas Y. Xia Z. Feng Q. (2016). A reference gene catalogue of the pig gut microbiome. *Nat. Microbiol.* 1:16161. 10.1038/nmicrobiol.2016.161 27643971

[B43] Yang H. Huang X. Fang S. He M. Zhao Y. Wu Z. (2017). Unraveling the Fecal Microbiota and Metagenomic Functional Capacity Associated with Feed Efficiency in Pigs. Front. Microbiol. *Front. Microbiol.* 8:1555. 10.3389/fmicb.2017.01555 28861066 PMC5559535

[B44] Yu C. Luo X. Zhan X. Hao J. Zhang L. Yb L. S. (2018). Comparative metabolomics reveals the metabolic variations between two endangered Taxus species (T. fuana and T. yunnanensis) in the Himalayas. *BMC Plant Biol.* 18:197. 10.1186/s12870-018-1412-4 30223770 PMC6142684

[B45] Zhao B. Wall R. J. Yang J. (2005). Transgenic expression of myostatin propeptide prevents diet-induced obesity and insulin resistance. *Biochem. Biophys. Res. Commun.* 337 248–255. 10.1016/j.bbrc.2005.09.044 16182246

[B46] Zhao L. Zhang Q. Ma W. Tian F. Shen H. Zhou M. (2017). A combination of quercetin and resveratrol reduces obesity in high-fat diet-fed rats by modulation of gut microbiota. *Food Funct.* 8 4644–4656. 10.1039/c7fo01383c 29152632

[B47] Zhu Z. Zhu B. Sun Y. Ai C. Wang L. Wen C. (2018). Sulfated Polysaccharide from Sea Cucumber and its Depolymerized Derivative Prevent Obesity in Association with Modification of Gut Microbiota in High-at Diet-ed Mice. *Mol. Nutrit. Food Res.* 62:e1800446. 10.1002/mnfr.201800446 30267558

