# Supplementary figures and images for: Integrated Microbiome and Metabolome Analysis Reveals a Positive Change in the Intestinal Environment of Myostatin Edited Large White Pigs
Source: Front Microbiol. 2021 Feb 17;12:628685. doi: 10.3389/fmicb.2021.628685 (PMC7925633; doi:10.3389/fmicb.2021.628685)

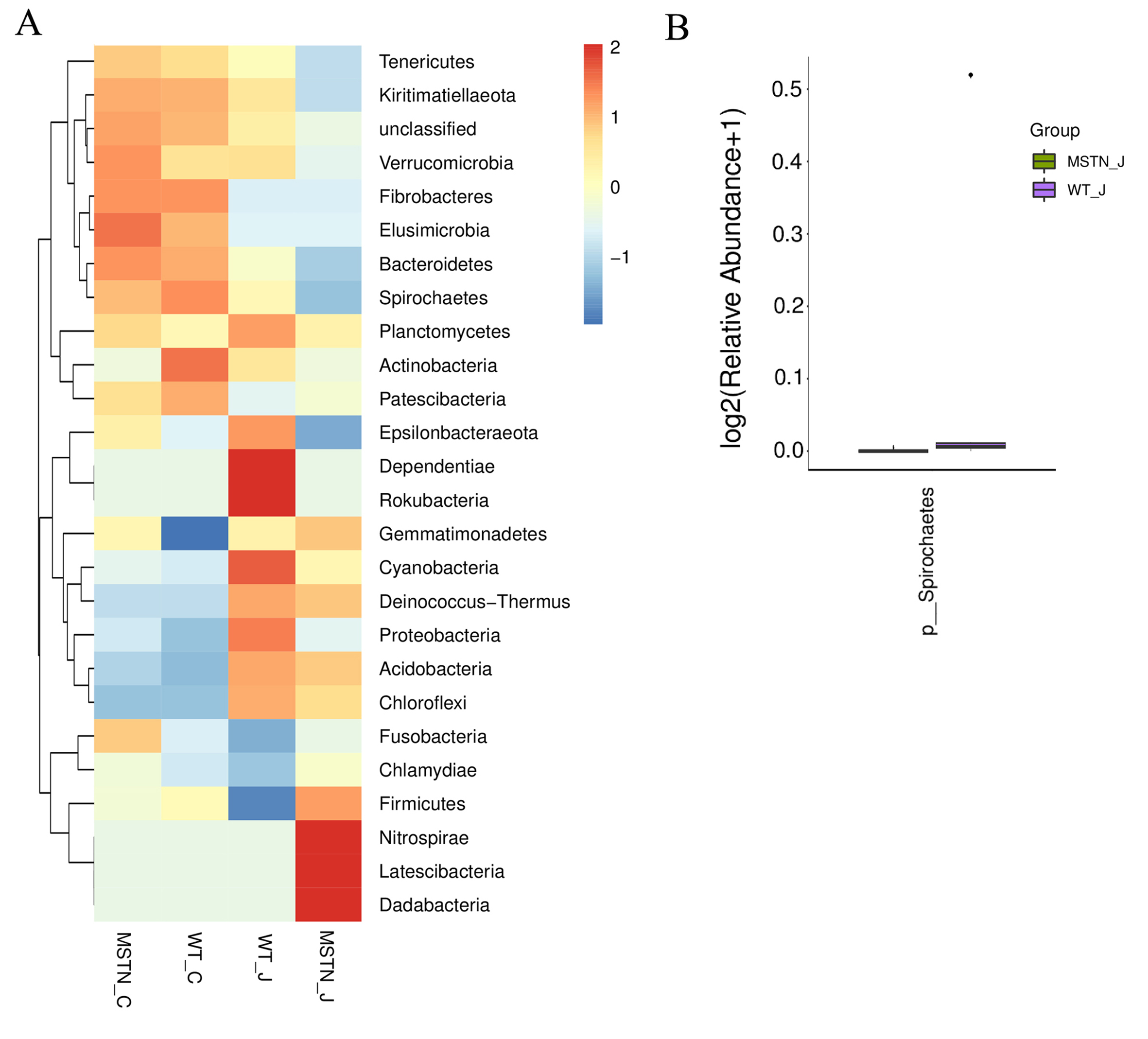

Supplement: Supplementary Figure 1 — Genotype identification results. (A) The mutation domain’s specific sequence of MSTN mutant large white pigs. (B) Genome sequencing results of heterozygote MSTN mutant and wild-type large white pigs. [file Image_1.JPEG]

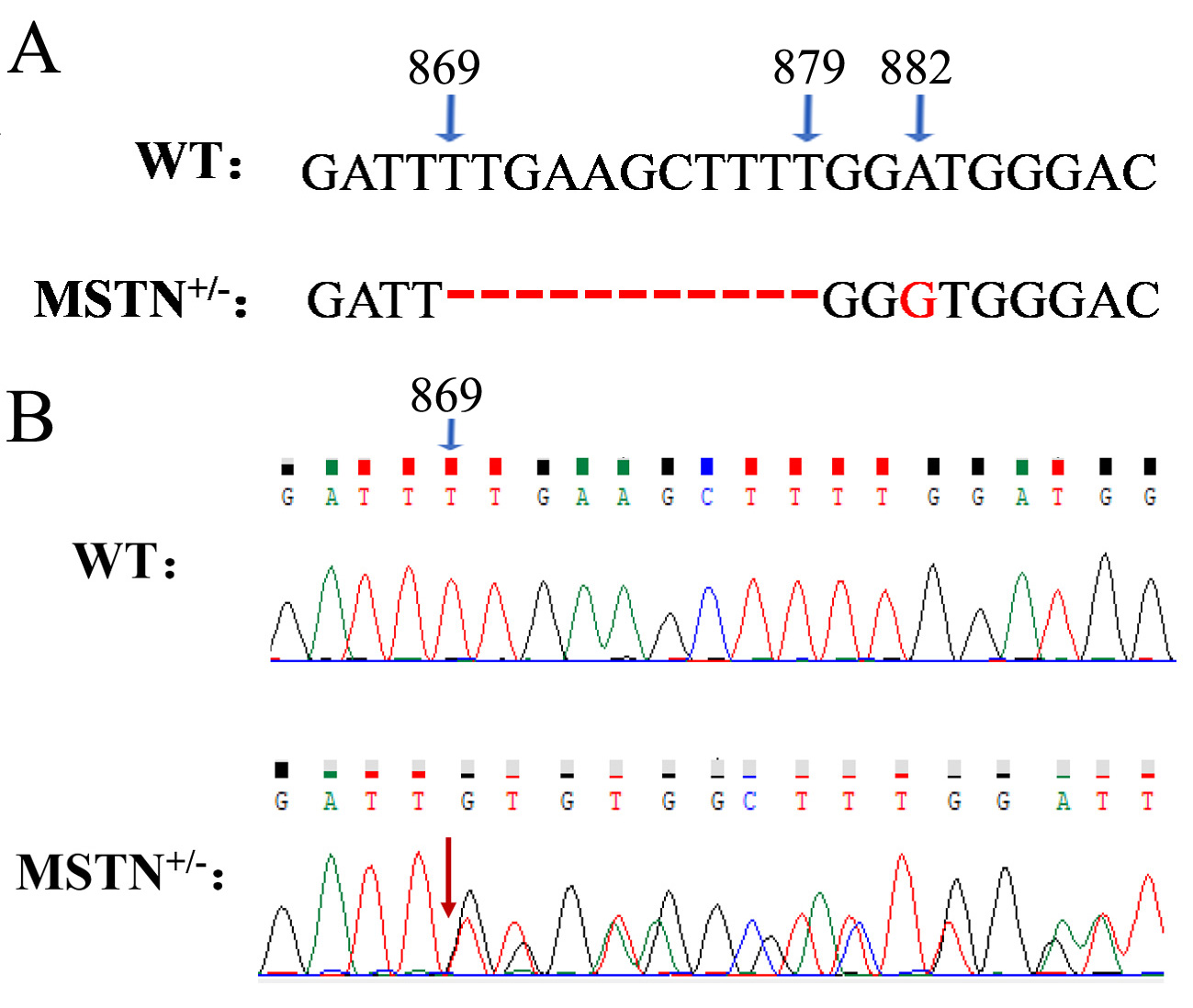

Supplement: Supplementary Figure 2 — Phylum differences between WT and MSTN groups. (A) Heat map showing the relative abundance of Phyla. (B) Significantly different genera in WT and MSTN jejunum samples. [file Image_2.JPEG]

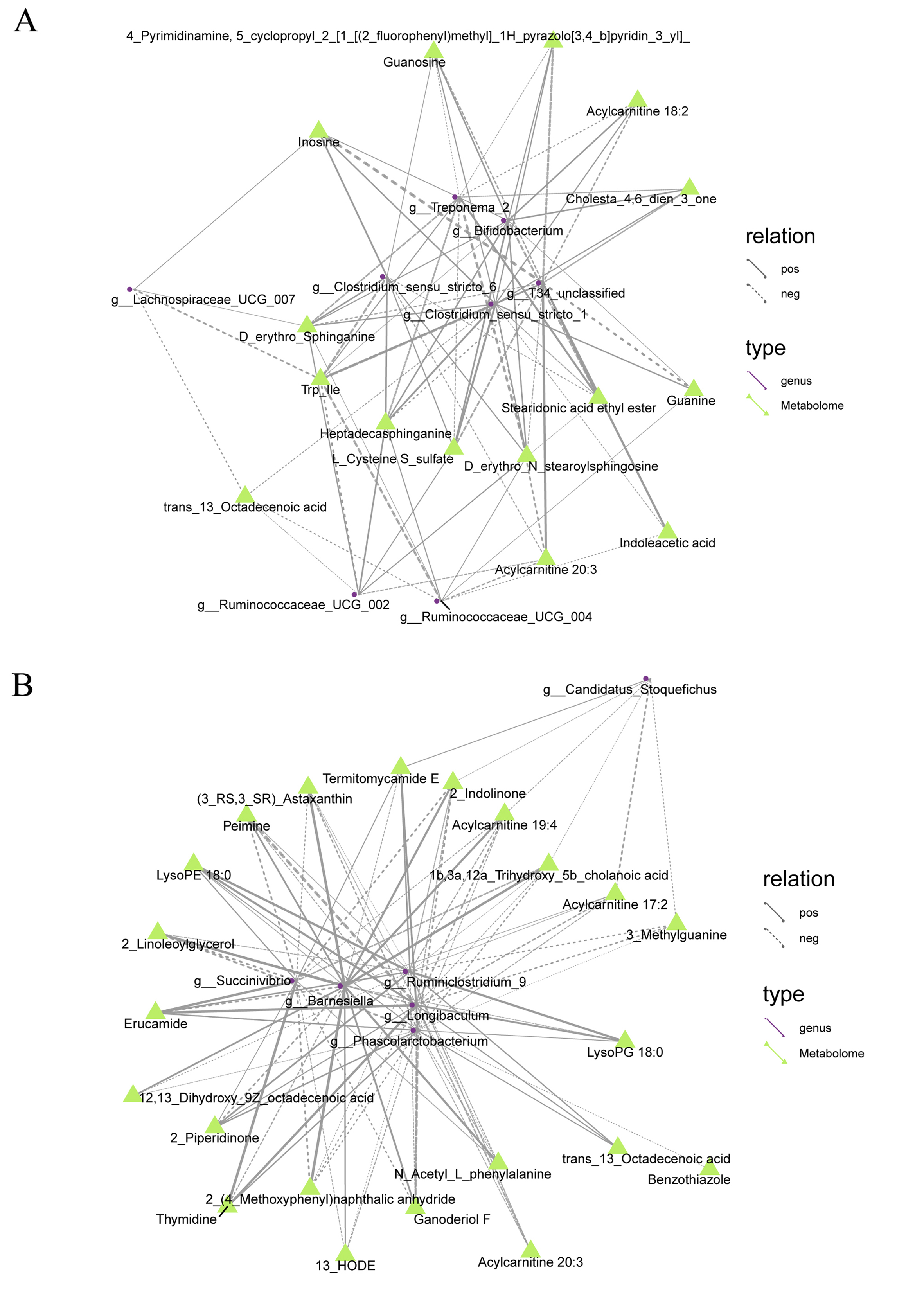

Supplement: Supplementary Figure 3 — Altered metabolites and genera network regulation analysis results. (A,B) Network regulation analysis results of altered metabolites and genera in the jejunum (A) and the cecum (B). The different nodes in the figure represent different bacterial communities or metabolites. Bacterial communities are represented by the round shapes while metabolites are represented by triangles. Solid and dashed lines represent the positive and negative correlations between the flora and the metabolites, respectively. [file Image_3.JPEG]
