# Supplementary material for: Integrated Microbiome and Metabolome Analysis Reveals a Positive Change in the Intestinal Environment of Myostatin Edited Large White Pigs
Source: Front Microbiol. 2021 Feb 17;12:628685. doi: 10.3389/fmicb.2021.628685 (PMC7925633; doi:10.3389/fmicb.2021.628685)
Supplement: Supplementary Table 1 — Information about of pigs in this study. [file Table_1.pdf]

Supplementary Table S1. The information of pigs.

	Gender	Breeds	Genotype	Father	Genotype	Breeds	Mother	Genotype	Breeds
1	Male	LW	Heter	6302	heter	LW	2305	WT	LW
2	Male	LW	Heter	6302	heter	LW	134612	WT	LW
3	Male	LW	Heter	6302	heter	LW	3217	WT	LW
4	Female	LW	Heter	6302	heter	LW	2305	WT	LW
5	Female	LW	Heter	6302	heter	LW	2305	WT	LW
6	Female	LW	Heter	6302	heter	LW	3217	WT	LW
7	Male	LW	WT	6302	heter	LW	3217	WT	LW
8	Male	LW	WT	6302	heter	LW	3217	WT	LW
9	Male	LW	WT	6302	heter	LW	3217	WT	LW
10	Female	LW	WT	6302	heter	LW	3217	WT	LW
11	Female	LW	WT	6302	heter	LW	2305	WT	LW
12	Female	LW	WT	6302	heter	LW	2305	WT	LW
