# Supplementary material for: Integrated Microbiome and Metabolome Analysis Reveals a Positive Change in the Intestinal Environment of Myostatin Edited Large White Pigs
Source: Front Microbiol. 2021 Feb 17;12:628685. doi: 10.3389/fmicb.2021.628685 (PMC7925633; doi:10.3389/fmicb.2021.628685)
Supplement: Supplementary Table 2 — Composition and nutrient levels of the diets (%, as-fed basis). [file Table_2.pdf]

Supplementary Table S2. Composition and nutrient levels of the diets (%, as-fed basis)

Calculated nutrient content	Percentage
Crude protein(%)	16
Crude ash(%)	8
Crude fiber (%)	1.98
Calcium （%）	0.6-0.9
Phosphorus（%）	0.5
Sodium chloride（%）	0.3-0.8
Lysine（%）	0.9
Water（%）	14
