# Supplementary material for: Integrated Microbiome and Metabolome Analysis Reveals a Positive Change in the Intestinal Environment of Myostatin Edited Large White Pigs
Source: Front Microbiol. 2021 Feb 17;12:628685. doi: 10.3389/fmicb.2021.628685 (PMC7925633; doi:10.3389/fmicb.2021.628685)
Supplement: Supplementary Table 3 — The R2 and Q2 values for all mathematical models. [file Table_3.pdf]

Supplementary Table S3. The R2 and Q2 values for all mathematical models

Mode	Comparison	P-value R2	P-value Q2	R2	Q2
pos	MSTN_J/WT_J	0.55	0.03	0.8883379	0.274810551
pos	MSTN_C/WT_C	0.25	0.015	0.9839648	0.353059477
neg	MSTN_J/WT_J	0.52	0.125	0.9155766	0.034364683
neg	MSTN_C/WT_C	0.11	0.015	0.9815042	0.388636497
